# Rv2617c and P36 are virulence factors of pathogenic mycobacteria involved in resistance to oxidative stress

**DOI:** 10.1080/21505594.2019.1693714

**Published:** 2019-11-29

**Authors:** Marina A. Forrellad, Cristina L. Vázquez, Federico C. Blanco, Laura I. Klepp, Elizabeth A. García, Rosana V. Rocha, Villafañe Luciana, María M. Bigi, Maximiliano G. Gutierrez, Fabiana Bigi

**Affiliations:** a Institute of Biotechnology, National Institute of Agricultural Technology (INTA, Instituto de Biotecnología, Instituto Nacional de Tecnología Agropecuaria) and IABIMO-National Scientific and Technical Research Council (CONICET, Consejo Nacional de Investigaciones Científicas y Tecnológicas), Hurlingham, Buenos Aires, Argentine; b University of Buenos Aires, School of Agronomy (Universidad de Buenos Aires, Facultad de Agronomía), Autonomous City of Bueno Aires, Argentine; c Host-pathogen interactions in tuberculosis laboratory, The Francis Crick Institute, London, UK

**Keywords:** *Mycobacterium tuberculosis*, oxidative stress, virulence factor, Rv2617c, Erp

## Abstract

In this study, we characterized the role of Rv2617c in the virulence of *Mycobacterium tuberculosis*. Rv2617c is a protein of unknown function unique to *M. tuberculosis* complex (MTC) and *Mycobacterium leprae. In vitro*, this protein interacts with the virulence factor P36 (also named Erp) and KdpF, a protein linked to nitrosative stress. Here, we showed that knockout of the *Rv2617c* gene in *M. tuberculosis* CDC1551 reduced the replication of the pathogen in a mouse model of infection and favored the trafficking of mycobacteria to phagolysosomes. We also demonstrated that Rv2617c and P36 are required for resistance to *in vitro* hydrogen peroxide treatment in *M. tuberculosis* and *Mycobacterium bovis*, respectively. These findings indicate Rv2617c and P36 act in concert to prevent bacterial damage upon oxidative stress.

## Introduction

*Mycobacterium tuberculosis* is the main causative agent of human tuberculosis (TB). According to the WHO, in 2017, 10 million people suffered from TB, whereas 1.6 million died from the disease (https://www.who.int/news-room/fact-sheets/detail/tuberculosis). Despite much progress has been made in the last decade, important aspects of the infection remain unknown. The slow decreasing of TB incidence in the world, the lack of an effective vaccine and the failure of the treatment of patients with XMDR tuberculosis are the most significant examples that illustrate the missing pieces of the complex puzzle that represents *M. tuberculosis* in its interaction with humans.

According to Mycobrowser (mycobrowser.epfl.ch) database, more than 4000 open reading frames have been annotated in the *M. tuberculosis* genome. To date, however, 1803 genes encoding proteins remain classified as unknown proteins and 68 of these unclassified proteins are putative transmembrane proteins. Rv2617c, which is only present among the members of the *M. tuberculosis* complex, is in this last group of proteins.

In a previous study, our group identified Rv2617c as a protein that interacts with P36, also called Mb3850, Erp or PirG. In turn, P36 is a virulence factor of *M. tuberculosis* and *Mycobacterium bovis* [1,2]. Olvera et al. have recently demonstrated that Rv2617c also interacts with KdpF, a protein linked to nitrosative stress [3].

The findings of the current study demonstrated that *M. tuberculosis* requires Rv2617c for a proper replication in a mouse model of tuberculosis and that this protein is relevant for phagosome maturation arrest induced by *M. tuberculosis*. Moreover, experimental evidence indicated that Rv2617c and P36 participate in the mechanism of resistance to oxidative stress, which is a strategy that *M. tuberculosis* displays to counteract the microbicidal action of the macrophages.

## Results

### The knockout of Rv2617c impaired the replication of M. tuberculosis in mice

A previous study reported that Rv2617c interacts with P36 protein in an *Escherichia coli* two-hybrid system and *in vitro* [4]. Indeed, P36 plays a role in the virulence of both *M. tuberculosis* [1] and *M.* bovis [2] and thus we evaluated the importance of Rv2617c in the replication of *M. tuberculosis* in a mouse model of infection.

**Figure 1.**
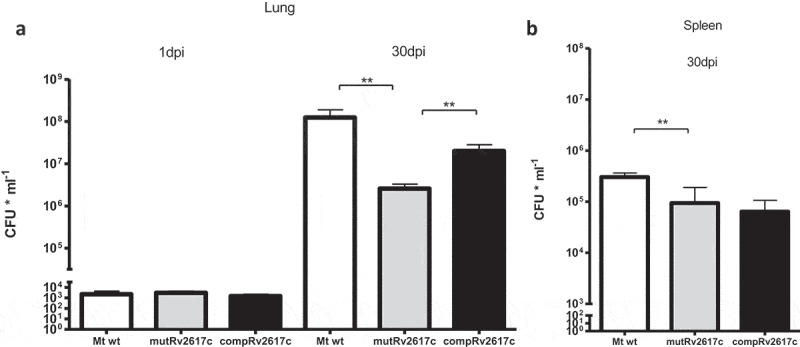
The knockout of *Rv2617c* impaired the replication of *M. tuberculosis* in mice. Groups of BALB/c mice were infected with *M. tuberculosis* CDC1551 (Mt wt), *Rv2617c* mutant (mutRv2617c) and complemented strain (comp). At 1 and 30 days post infection (dpi) the mice were sacrificed; the lungs (a) and spleens (b) were extracted and homogenized to assess CFU (expressed as CFU ml^−1^) on solid medium. The values are expressed as the mean ± S.D. of CFUs for six mouse organs. The data were analyzed using t-test (**p < 0.01). This experiment was performed once.

We determined the bacterial loads in lung and spleen of mice that had been intratracheally infected with 10^3^ bacteria of the wild type *M. tuberculosis* CDC1551, mutant MtbRv2617c::Kn (mutRv2617c) or complemented strain mutRv2617c/pVV16::Rv2617c. The bacterial load in lungs of mice infected with mutRv2617c was significantly lower than that of the mice infected with the wild type (Mt wt) or complemented strains (Figure 1(a)). The bacterial replication in spleen was also significantly impaired in the mutant strain; however, complementation with an intact copy of *Rv2617c* gene in the mutant strain failed to restore the wild type virulence (Figure 1(b)). The multi-copy nature of the complementing plasmid may interfere with the normal bacterial growth and this may be the cause of the lack of complementation. Indeed, in the study of Sassetti et al. [5] *Rv2617c* was among the genes whose inactivation was advantageous for the *in vitro* growth of *M. tuberculosis*. The growth of the complemented strain displayed a delay of 7 to 10 days in solid medium in comparison to that of the wild type and mutant strains (data not shown).

Thus, these results indicate that Rv2617c is essential for the full replication of *M. tuberculosis* in the lungs of mice.

### The lack of Rv2617c affects the phagosomal modulation induced by M. tuberculosis inside host cells

One of the main mechanisms that *M. tuberculosis* displays to counteract the microbicidal response of the macrophages is to modulate the maturation of the phagosome that contain them. In this way, mycobacteria avoid the trafficking to more aggressive endosomal compartments such as phagolysosomes [6].

To evaluate whether the reduced replication of mutRv2617c could be associated to a lower capacity of the mutant strain to modulate phagosomal function, we analyzed the intracellular trafficking of the wild type, mutant and complemented strains by immunofluorescence and confocal microscopy. For this purpose, we analyzed and quantified the association of the late endosomal marker LAMP-2 to the phagosome containing the *M. tuberculosis* strains after 3 h of infection in J774 macrophages.

**Figure 2.**
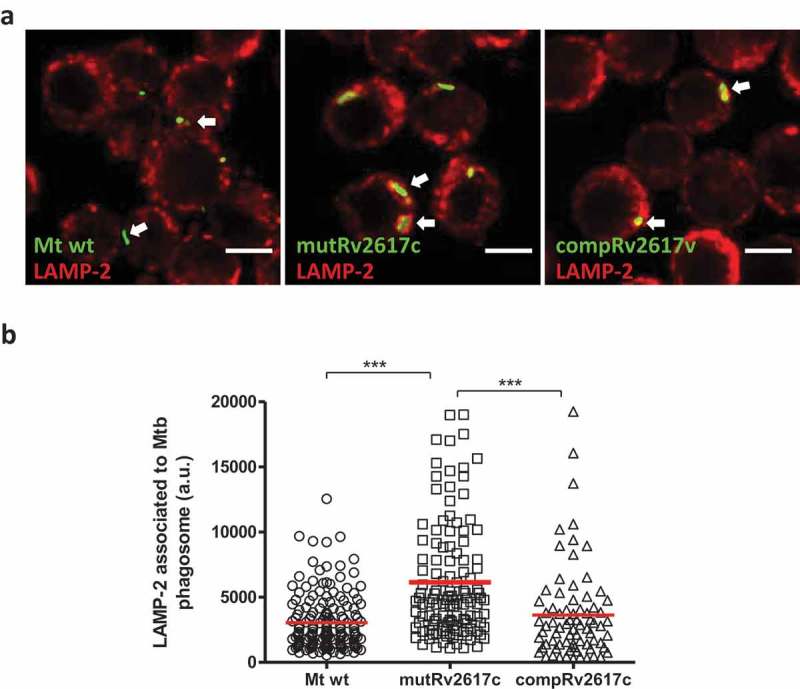
The lack of Rv2617c impaired the modulation of phagosomal maturation induced by *M. tuberculosis* inside murine macrophages. Confocal microscopy analysis of murine J774 macrophages infected with *M. tuberculosis* CDC1551 (Mt wt), *Rv2617c* mutant (mutRv2617c) and complemented (compRv2617c) strains. A) Representative images of J774 macrophages infected with the different strains labeled with FITC (green fluorescence). The cells were fixed after 3 h of infection and the late phagosomal marker LAMP-2 were detected by indirect immunofluorescence using a specific anti-LAMP-2-Cy5 (red fluorescence). Scale bars: 10 μm. Merge indicates the location of the different strains in the phagolysosome. B) Fluorescence intensity quantification of LAMP-2 associated with phagosome containing the wild type (Mt wt, circles), mutant, (mutRv2617c, squares) or complemented (compRv2617c, triangles) strains expressed as arbitrary units (a.u.). Fiji software was used for quantification. The values are representative of three independent experiments with similar results. The data were analyzed using one-way ANOVA analysis and Bonferroni’s posttest (***p < 0.001).

Strain mutRv2617c associated significantly more (p ≤ 0.001) with LAMP-2 than the wild type and complemented strains (Figure 2(a,b)). This is consistent with a reduced ability to modulate phagosome maturation. Replication assays in J774 macrophages displayed similar number of colony forming units (CFU) of the mutant and wild type strains at 48 and 72 h post infection (data not shown).

These results suggest that Rv2617c participates in the mechanism of phagosomal maturation at early time of infection. However, this role is insufficient to avoid the microbicidal attack later during infection.

### Rv2617c is essential for M. tuberculosis resistance to oxidative stress

The *in silico* analysis of Rv2617c sequence revealed that this protein has some similarity to the DoxD subunit of the terminal quinol:oxygen oxidoreductase complex of *Acidianus ambivalen* (Supplementary material S1). This protein complex consists of four subunits (DoxA, DoxB, DoxC and DoxD) and is probably involved in the electron transport that occurs during aerobic sulfur oxidation in *A. ambivalens* [7].

**Figure 3.**
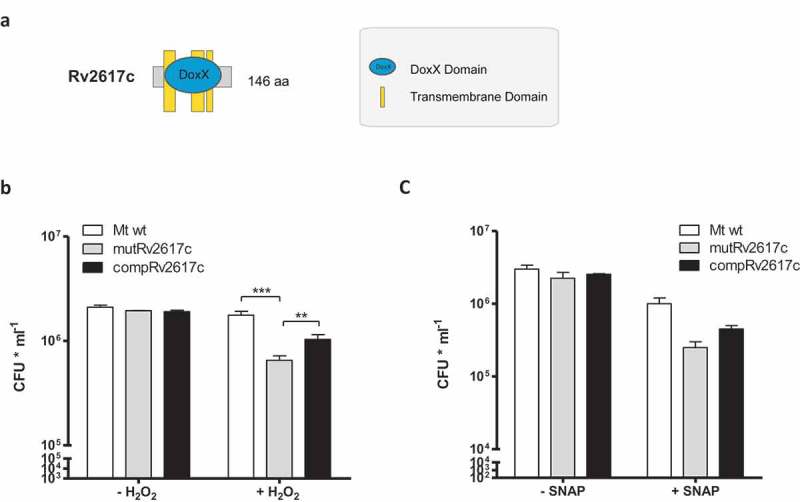
Rv2617c is necessary for *M. tuberculosis* resistance to oxidative stress. (a) Schematic representation of DoxX domain in Rv2617c. (b,c) Bacterial viability after exposure to 10 mM H_2_O_2_ (b) and 1 mM SNAP (c) for 3 h. Bacterial strains were treated or not with the reagents as described in Materials and Methods and dilutions of cultures were plated on solid medium for CFU counting. The data show means of CFU ± standard deviations of triplicates and are representative of four independent experiments. The statistical analysis was performed using one-way ANOVA and Bonferroni’s posttest (**p < 0.01, ***p < 0.001).

Nambi and collaborators have delineated an oxidative stress network of *M. tuberculosis* through genetic interaction maps of *ctpC*, a gene encoding a P-type ATPase responsible for metalating SodA with Mn^2+^[8], with all *M. tuberculosis* genes with predictable functions [9]. In their study, they found genetic interaction between the *ctpC* and *Rv3005c*, among other genes, during mouse infection [9]. They also demonstrated that Rv3005c, SodA and Sse (a predicted thiol-oxidoreductase) form a membrane-associated oxidoreductase complex [9]. Interestingly, *Rv3005c* encodes an integral membrane protein that carries a DoxX domain that is also present in Rv2617c (Figure 3(a)).

Based on these observations, we subsequently investigated the role of Rv2617c in the resistance to oxidative stress of *M. tuberculosis* by comparing the survival of the mutRv2617c to that of the wild type upon oxidative stress. For this purpose, we incubated the different strains in 10 mM H_2_O_2_ for 3 and 16 h and counted the surviving bacteria on solid medium. Upon 3 h of H_2_O_2_ treatment, the CFU of the wild type and complemented strains were significantly higher than that of the mutant strain (Figure 3(b)). Longer hydrogen peroxide treatment (16 h) severely affected the growth of all strains (Supplementary material S2).

Rv2617c is a potential integral membrane protein and therefore the lack of this protein could alter the normal membrane structure and protein secretion and in turn may increase the sensitivity to stresses. However, we found that the cellular and culture supernatant protein profiles of mutRv2617c were similar to those of wild type strain. Moreover, western blot experiments identified similar amounts of P36, P27 (LprG) and Ag85B proteins in the culture supernatant fractions of the wild type and mutant strains (Supplementary material S3)”.

Altogether, these results indicate that Rv2617c plays a role in the resistance of *M. tuberculosis* to oxidative stress *in vitro*.

Olvera et al. [3] as well as Ohno et al. [10] have provided evidence supporting the idea that Rv2617c participates in the mechanisms of response to nitrosative stress of *M. bovis* BCG and *M. tuberculosis*. In this study, we assessed and compared the resistance of the mutant Rv2617c upon a nitrosative stress with that of the complemented and wild type strains. For this purpose, we induced nitrosative stress by exposing the bacteria with S-nitroso-N-acetyl-D, L-penicillamine (SNAP), which is a nitric oxide (NO) donor. This compound induces nitrosative stress and therefore upregulates the expression of Rv2617c in *M. bovis* BCG [3].

The survival of mutant Rv2617c upon incubation with 1 mM SNAP for 3 h was reduced, although not significantly, in comparison to that of the wild type and complemented strains (Figure 3(c)). Incubation of bacterial strains with diethylenetriamine, a nitric oxide adduct, retrieved similar results (100 mM DETA/NO) (Supplementary material 4).

Thus, these results suggest that either *M. tuberculosis* compensates in some way the lack of Rv2617c to resist nitrosative stress or that Rv2617c plays a minor role during this response mechanism.

**Figure 4.**
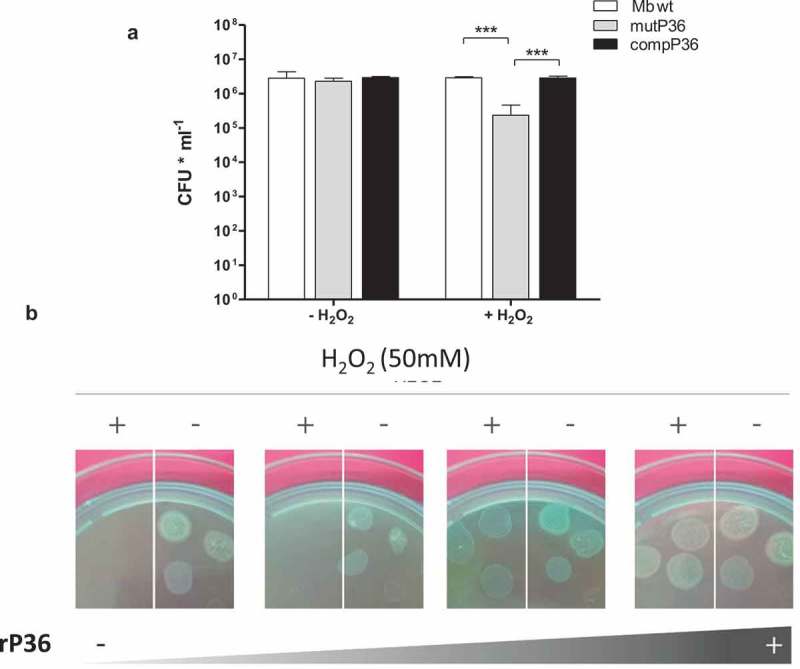
P36 is necessary for *M. bovis* resistance to oxidative stress. (a) Bacterial viability after 16 h exposure to 10 mM H_2_O_2_. The data show means of CFU ± standard deviations of triplicates and are representative of four independent experiments. The statistical analysis was performed using t- test (*** p < 0.001). (b) MutP36 incubated 16 h with 0, 4, 20 and 60 µg/ml of rP36 recombinant protein in the presence or in the absence of 50 mM H_2_O_2_.

We have previously shown that Rv2617c interacts with the *M. bovis* virulence factor P36 *in* *vitro* and *in* *vivo* in a two-hybrid system [4]. To determine whether P36 is functionally connected with Rv2617c, we compared the resistance to hydrogen peroxide of a *M. bovis* mutant strain in *P36* gene (mutP36 [1]) to that of its parental and complemented strains. Incubation in the presence of 10 mM of H_2_O_2_ for 3 and 16 h produced no variation of the CFU of the wild type and complemented strains in comparison to the control condition (without hydrogen peroxide); the CFU of mutP36, by contrast, significantly diminished upon oxidative stress (Figure 4(a) and S5).

P36 is a secreted protein with a probable localization in the cell wall. Thus, to confirm the role of P36 in oxidative stress resistance, we subsequently incubated mutP36 cultures with increasing concentrations of recombinant P36 (rP36) with or without 50 mM H_2_O_2_. Again, the addition of rP36 protein to mutP36 cultures reverted the sensitivity of the mutant strain to H_2_O_2_ in a dose-dependent manner (Figure 4(c)).

## Discussion

Resistance to macrophage-mediated killing is critical to the virulence of pathogenic mycobacteria. Upon phagocytosis of bacteria, macrophages produce reactive oxygen species (ROS) and reactive nitrogen species (RNS) with potential bactericidal activity. These species react with many molecules, including nucleic acids, proteins, lipids and carbohydrates. Most intracellular pathogens, including mycobacterial species, have developed different defense strategies to protect themselves against the damaging effects of these agents [6].

Pathogenic mycobacteria display several strategies to counterattack oxidative burst. For example, some mycobacteria produce mycothiol as a relevant redox buffering system and antioxidant enzymes, including AhpC, SodC, SodA, KatG and TpX [6]. Apart from the proteins that directly participate in the ROI detoxification mechanisms, other proteins with non-conserved detoxification activities confer protection against oxidative burst. Among these non-classical oxidative stress response proteins, there are enzymes involved in the cell wall integrity, such as Rv2136c, Rv2445c and PonA2 [6], as well as proteins that have functional relationships with antioxidant enzymes, such as DoxX (Rv3005c) and SseA [9]. DoxX and SseA together with SodA form an oxidoreductase complex in *M. tuberculosis*. SseA is a predicted thiol-oxidoreductase and DoxX is an integral membrane protein that carries the DoxX domain present in sulfur oxidation proteins of *archea* [9].

The results of this study suggest that Rv2617c, another *M. tuberculosis* protein with DoxX domain, and the virulence factor P36 play a role in the mycobacterial machinery implicated in the resistance to oxidative burst. Consistent with our findings, Mehra and Kaushal [11] have shown that P36 is under the control of sigma factor H (SigH) and that P36 expression is upregulated upon oxidative stress induced by a treatment with diamide for 30 min. Therefore, *P36* seems to be part of the initial transcriptional program under the control of SigH that responds to oxidative stress [11].

One interesting outcome of this work is the higher resistance of *M. bovis* to hydrogen peroxide in relation to *M. tuberculosis*. Further studies should address the implications of these differential oxidative stress resistant phenotypes in the interactions with the hosts and extend the analysis to other mycobacterial strains.

Both Rv2617c and P36 are essential for full virulence of *M. tuberculosis* and *M. bovis*, respectively, in mice [1,2]. Thus, Rv2617c and P36 may be necessary for oxidative stress resistance during host infections. Indeed, a *M. tuberculosis* mutant in *erp* gene, the homolog of *P36* in *M. bovis*, displayed an impaired replication inside macrophages [2]. Furthermore, in the present study the lack of Rv2617c partially delayed phagosome maturation during *M. tuberculosis* cell host infection. The mutant lacking Rv2617c, however, showed similar replication levels in mouse macrophages than the wild type strain (data not shown). Probably, the full intracellular function of Rv2617c requires an IFNγ-mediated macrophage activation.

Future studies will delineate more precisely how Rv2617c and P36 contribute to protect pathogenic mycobacteria from oxidative stress.

## Materials and methods

### Bacterial strains and culture media

*M. tuberculosis* CDC 1551 (Mt wt), mutRv2617c and compRv2617c strains were grown in Middlebrook 7H9 medium (Difco Laboratories, 271310) supplemented with albumin 0.5%, dextrose 0.4% and 0.5% glycerol (M7H9-AD-G) or Middlebrook 7H10 (Difco Laboratories, 262710) supplemented with AD-G (7H10-AD-G). Albumin was omitted from the medium when preparing culture supernatant proteins. *M. bovis* NCTC10772 (Mb wt), mutP36 and compP36 strains were grown in M7H9-AD supplemented with 0.5% pyruvate (M7H9-AD-P) or 7H10-AD-P. When necessary, either 50 µg/ml hygromycin or 20 µg/ml kanamycin or Tween 80 (T80) 0.05% was added to the media.

The strain mutRv2617c is a *M. tuberculosis* CDC 1551 strain with a *Himar1* transposon insertion in *Rv2617c* gene [12]. The lack of an intact *Rv2617c* gene in mutRv2617c was confirmed by PCR analysis (data not shown). MutRv2617c was complemented with the plasmid pVV16-Rv2617c, a replicative plasmid carrying *Rv2617c* inserted in *NdeI* and *HindIII* restriction sites (compRv2617c).

The infection and nitrosative stress experiments consisted of culturing the *M. tuberculosis* strains up to late exponential growth phase to subsequently harvest, wash and resuspend them in phosphate buffer saline (PBS) 1X. The bacterial suspensions were passed through a syringe needle (25 gauge) 30 times to disaggregate bacterial clumps. The remaining clumps were separated from individual bacteria by centrifugation at 900 g for 8 min. The nitrosative stress experiments were developed by resuspending the disaggregated bacterial cells in 1 mM SNAP (Sigma-Aldrich, N3398). The oxidative stress experiments were carried out by culturing mycobacterial strains up to late exponential growth phases to subsequently centrifuge the bacteria at 1000 g for 7 min in order to eliminate bacterial clumps. Disaggregated bacterial were incubated with 10–50 mM H_2_O_2_. After incubation, the bacterial cells were diluted in PBS and serial dilutions were plated on solid medium for CFU counting.

The recombinant P36 protein was produced and purified from *Escherichia coli* as previously described [4].

### Mouse infection

BALB/c mice (6–8 weeks of age) were infected with a suspension of *M. tuberculosis* CDC 1551 (wild type), the mutRv2617c or the complemented strain at an infective dose of 1*10^3^ bacteria per mouse suspended in 100 μl of PBS buffer. The strains were administered intratracheally as described previously [13]. At 1 and 30 days post infection, mice were sacrificed and the lungs and spleen were extracted. The organs were homogenized and plated on solid medium to determinate the CFUs.

These experiments were performed in compliance with the regulations of the Institutional Animal Care and Use Committee (CICUAE) of INTA.

### Indirect immunofluorescence and confocal microscopy

The *M. tuberculosis* strains were covalently stained with fluorescein isothiocyanate (FITC) (Sigma-Aldrich, 3326-32-7) as previously described [14]. The stained bacteria were used to infect J774 murine macrophages at a MOI of 5:1 for 1 h at 37°C and 5% CO_2_ (uptake). The infected macrophages were washed to eliminate the extracellular bacteria and incubated again for 2 h (chase). Indirect immunofluorescence was performed on the infected cells as previously described [14], using anti-LAMP-2 (1/50 diluted in PBS). Anti-rat antibody conjugated to Cy5 (Indodicarbocyanine, Jackson ImmunoResearch Inc.) was used as secondary antibody. The cells were analyzed by confocal microscopy using a Leica TCS-SP5 spectral confocal microscope (Leica Microsystems) at the integrated Microscopy laboratory (UNLP, CICVyA, INTA, Argentina). Mycobacterial internalization was analyzed and quantified using Fiji software (U.S. National Institutes of Health, Bethesda, MD) as described previously [14]. Each experiment was performed in duplicates.

Statistical analysis was performed using analysis of variance (ANOVA) and Bonferroni’s posttests. Three independent infections were performed for each assay.

Fluorescence intensity values were plotted and analyzed using GraphPad Prism 5 (GraphPad Software, Inc.).

### Protein sample preparations and SDS-PAGE

Mycobacterial cells were harvested by centrifugation, disrupted with glass beads in a homogenizer, centrifuged at 1000 g for 10 min at 4°C and supernatants were collected for protein quantification. The culture supernatants containing secreted proteins were treated with 10% trichloroacetic acid overnight and centrifuged at 7000 g for 30 min at 4°C. Protein pellets were neutralized with 1M Tris (Sigma).

Protein samples from culture supernatants and cells were resuspended in cracking buffer (60 mM Tris-Cl pH 6.8, 2% SDS, 10% glycerol, 2% β-mercaptoethanol, 0.01% bromophenol blue), boiled for 4 min and resolved in 12% SDS-PAGE. The visualization of the proteins was achieved by staining the gels with Coomassie blue.

Western blot assays were performed as previously described [15] using α-P36 (1/300) and α-P27 (1/300) monoclonal antibodies and α-Ag85 (1/250) rabbit polyclonal antibody as primary antibodies.
