# Supplementary material for: Rv2617c and P36 are virulence factors of pathogenic mycobacteria involved in resistance to oxidative stress
Source: Virulence. 2019 Nov 29;10(1):1026–33. doi: 10.1080/21505594.2019.1693714 (PMC6930017; doi:10.1080/21505594.2019.1693714)
Supplement: Supplemental Material [file kvir-10-01-1693714-s001.pptx]

## Slide 1
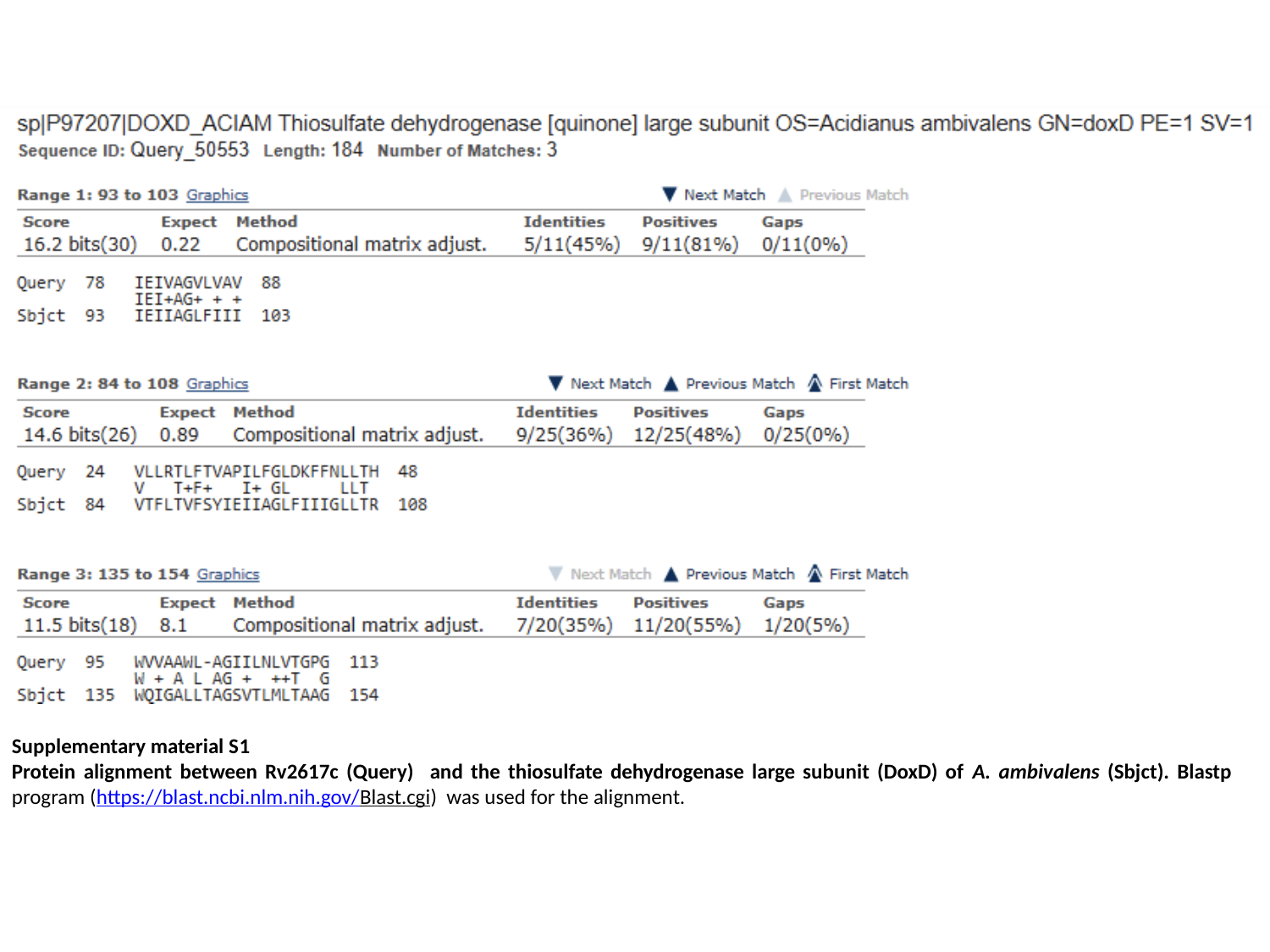

Supplementary material S1
Protein alignment between Rv2617c (Query) and the thiosulfate dehydrogenase large subunit (DoxD) of A. ambivalens (Sbjct). Blastp program (https://blast.ncbi.nlm.nih.gov/Blast.cgi) was used for the alignment.

## Slide 2
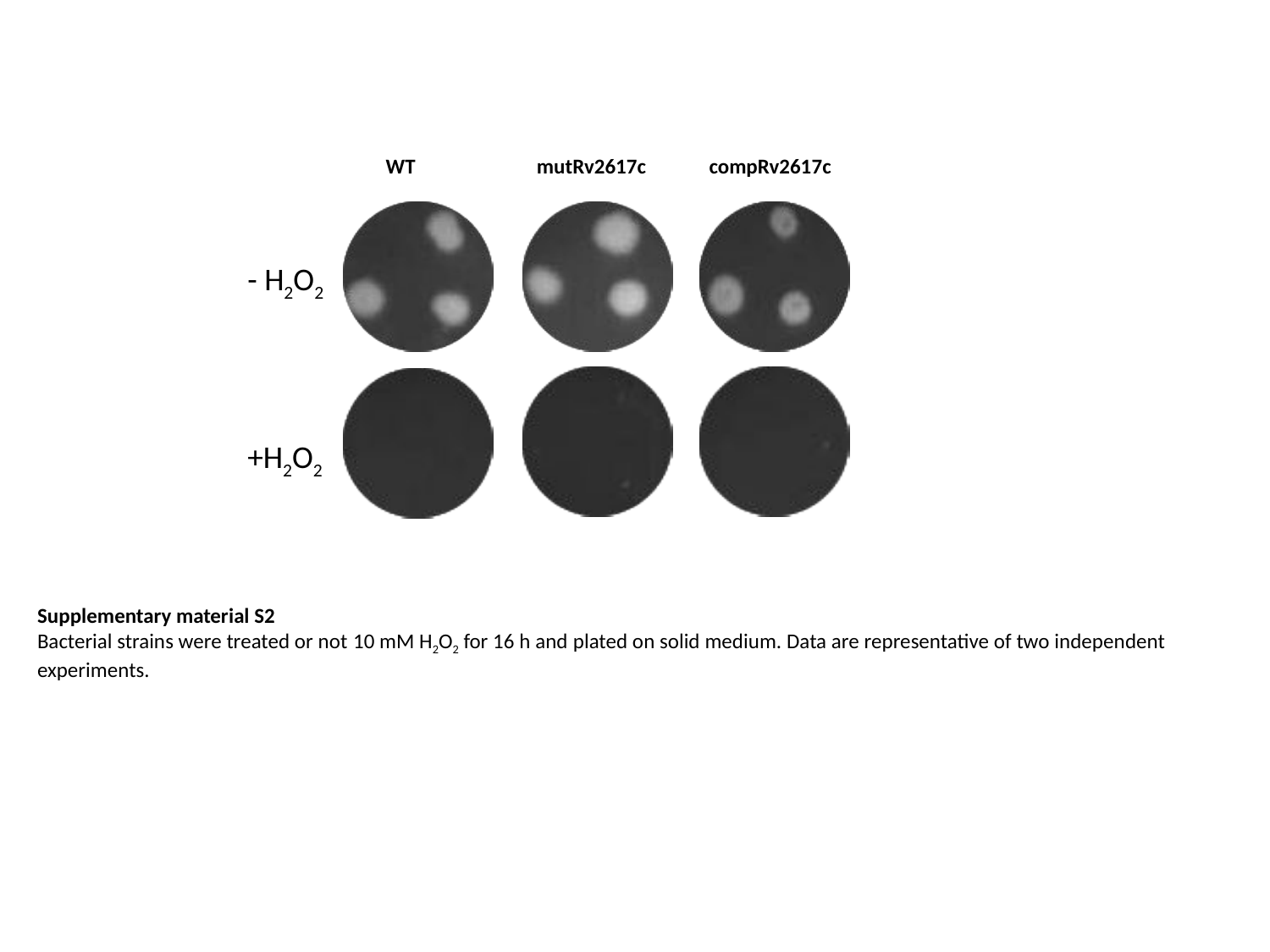

WT mutRv2617c compRv2617c
- H2O2
+H2O2
Supplementary material S2
Bacterial strains were treated or not 10 mM H2O2 for 16 h and plated on solid medium. Data are representative of two independent experiments.

## Slide 3
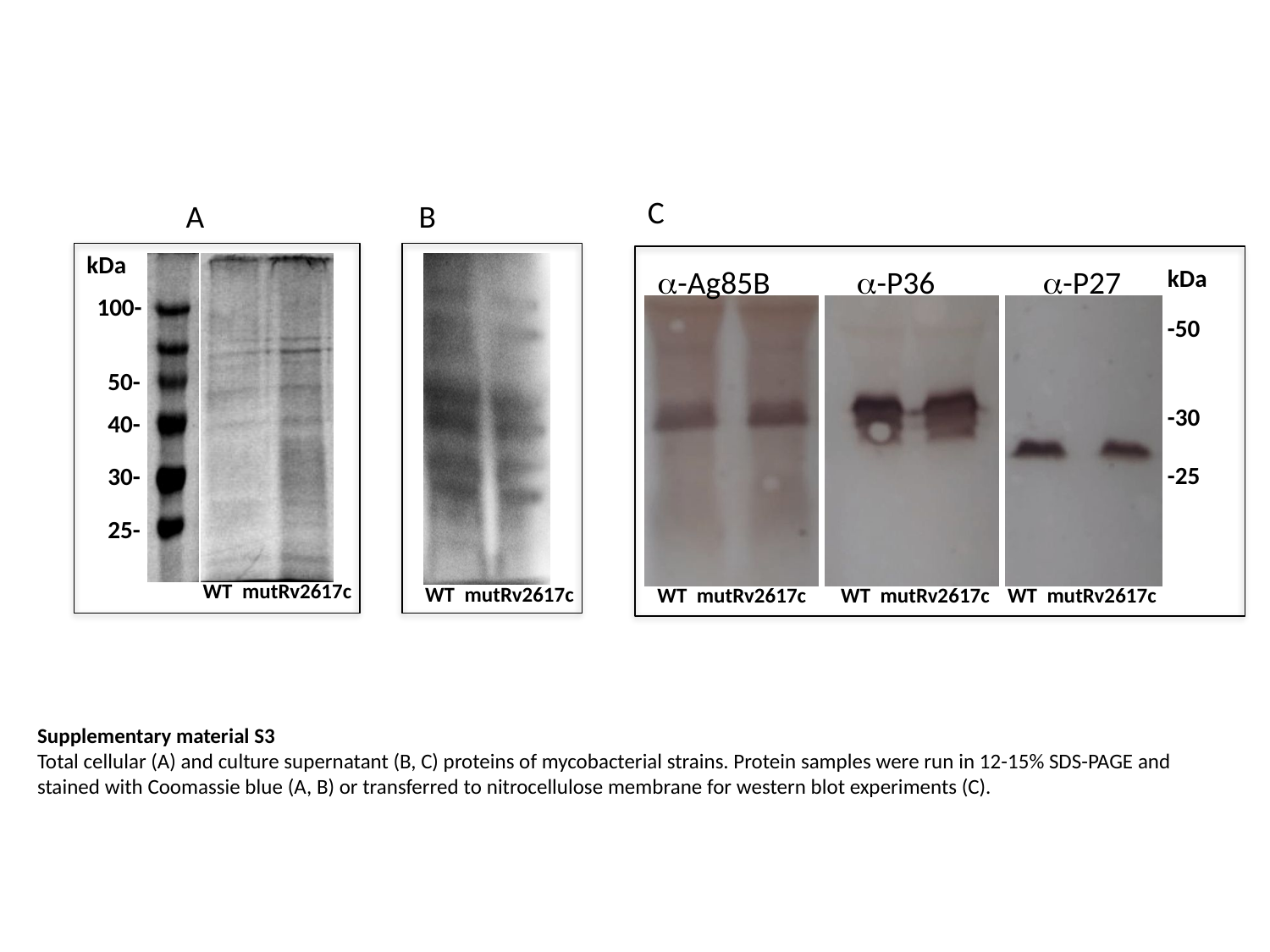

C
A
B
kDa
a-Ag85B a-P36 a-P27
kDa
100-
-50
-30
-25
50-
40-
30-
25-
WT mutRv2617c
WT mutRv2617c
WT mutRv2617c
WT mutRv2617c
WT mutRv2617c
Supplementary material S3
Total cellular (A) and culture supernatant (B, C) proteins of mycobacterial strains. Protein samples were run in 12-15% SDS-PAGE and stained with Coomassie blue (A, B) or transferred to nitrocellulose membrane for western blot experiments (C).

## Slide 4
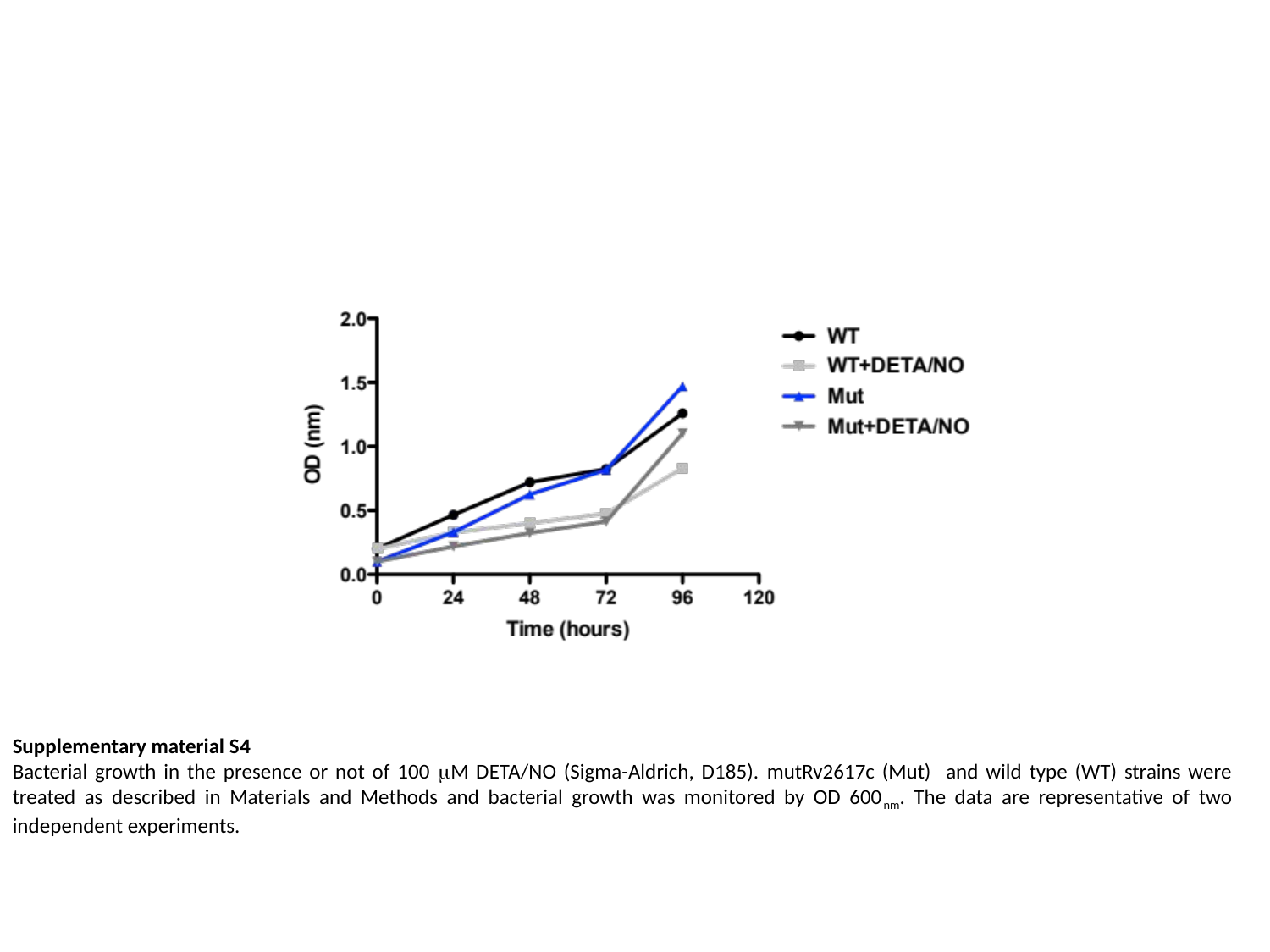

Supplementary material S4
Bacterial growth in the presence or not of 100 mM DETA/NO (Sigma-Aldrich, D185). mutRv2617c (Mut) and wild type (WT) strains were treated as described in Materials and Methods and bacterial growth was monitored by OD 600nm. The data are representative of two independent experiments.

## Slide 5
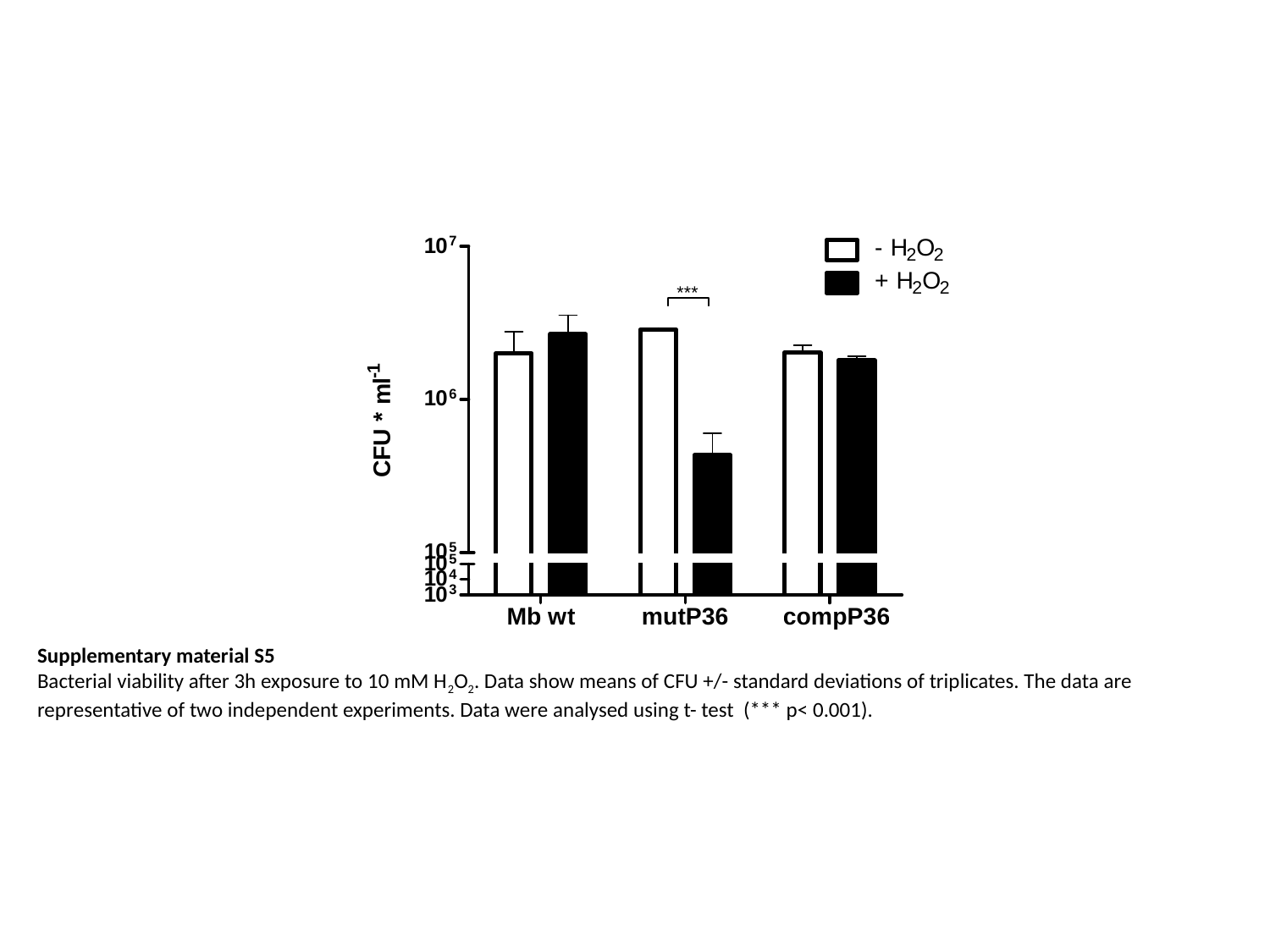

Supplementary material S5
Bacterial viability after 3h exposure to 10 mM H2O2. Data show means of CFU +/- standard deviations of triplicates. The data are representative of two independent experiments. Data were analysed using t- test (*** p< 0.001).
